# Prevalence of postpartum depression and antenatal anxiety symptoms during COVID-19 pandemic: An observational prospective cohort study in Greece

**DOI:** 10.18332/ejm/146233

**Published:** 2022-04-13

**Authors:** Georgia Micha, Thomas Hyphantis, Chryssoula Staikou, Dimitrios Valsamidis, Eleni Arnaoutoglou, Petros Tzimas, Nikolaos Vlahos, Alexandros Daponte, Ioannis Grypiotis, Polyxeni Pappa, Erofili Evangelaki, Sofia Apostolidou, Vasileios Paschos, Giolanda Varvarousi, Metaxia Bareka, Gloria E. Izountouemoi, Orestis Tsonis, Iouliani Koullourou, Konstantina Kalopita, Konstantinos Kotsis

**Affiliations:** 1 Department of Anesthesiology, “Helena Venizelou” General and Maternity Hospital of Athens, Athens, Greece; 2 Department of Psychiatry, Faculty of Medicine, School of Health Sciences, University of Ioannina, Ioannina, Greece; 3 1st Department of Anesthesiology, Aretaieion Hospital, National and Kapodistrian University of Athens, Athens, Greece; 4 Department of Anesthesiology, Alexandra General Hospital of Athens, Athens, Greece; 5 Department of Anesthesiology, Faculty of Medicine, School of Health Sciences, University of Thessaly, Larissa, Greece; 6 Department of Anesthesiology and Postoperative Intensive Care, Faculty of Medicine, School of Health Sciences, University of Ioannina, Ioannina, Greece; 7 2nd Department of Obstetrics and Gynecology, Aretaieion Hospital, National and Kapodistrian University of Athens, Athens, Greece; 8 Department of Obstetrics and Gynecology, Faculty of Medicine, School of Health Sciences, University of Thessaly, Larissa, Greece; 9 Department of Obstetrics and Gynecology, University Hospital of Ioannina, University of Ioannina, Ioannina, Greece; 10 Mental Health Center, “G. Hatzikosta” General Hospital of Ioannina, Ioannina, Greece

**Keywords:** postpartum depression, COVID-19, Edinburgh Postnatal Depression Scale (EPDS), anxiety

## Abstract

**INTRODUCTION:**

A significant proportion of pregnant women and women in the early postpartum period suffer from mental health problems. The COVID-19 pandemic represents a unique stressor during this period and many studies across the world have shown elevated rates of postpartum depression (PPD).

**METHODS:**

In this multicenter two-phase observational prospective cohort study, we aim to assess the prevalence of anxiety prior to labor (Generalized Anxiety Disorder-7), as well as PPD at 6–8 weeks postpartum using the Edinburgh Postnatal Depression Scale (EPDS).

**RESULTS:**

Of the 330 women analyzed, 13.2% reported symptoms of depression using EPDS cut-off score ≥13. High antenatal levels of anxiety (24.8% scored ≥10 in GAD-7) were documented. A significant proportion of postpartum women reported a decrease in willingness to attend antenatal education courses (36%) and fewer antenatal visits to their obstetrician (34%) due to pandemic. Higher antenatal anxiety increased the odds of being depressed at 6–8 weeks postpartum (EPDS ≥13).

**CONCLUSIONS:**

Compared to reported prevalence of PPD from previous studies before the COVID-19 era in Greece, we did not find elevated rates during the first wave of the pandemic. High anxiety levels were observed indicating that there is a need for close monitoring in pregnancy during the pandemic and anxiety screening to identify women who need support in the pandemic era. A well-planned maternity program should be employed by all the associated care providers to maintain the proper antenatal care adjusted to the pandemic strains as well as a follow-up after labor.

## INTRODUCTION

COVID-19 viral pandemic has imposed a great worldwide psychological burden attributed to the fear of wellbeing and to an immediate threat to human life per se. The SARS-CoV-2 virus is associated with profound psychological implications ranging from stress and generalized anxiety disorder to depression and suicidal tendencies^1,2^. This is not unprecedented since there is previous experience of mental onus in recent outbreaks^3^ and all clinical manifestations are exaggerated during lockdown periods, by the fear of forthcoming socioeconomic crisis and loss of employment^4^.

Pregnancy is a period of conflicting emotions ranging from joyful expectations of the forthcoming baby to the fear of childbirth, trait anxiety and distress over the couple’s adjustment, while diagnosis of clinical depression intensifies the previous conditions^5-7^. In the context of the COVID-19 pandemic, emotions of fear seem to be more prevalent (49%) than before (7.5%) and joyful expectations are overclouded by the pandemic (63% before vs 17% after)^8^.

COVID-19-related concerns surfaced because this viral infection is considered to be a state of increased risk of severe illness, as was reported in previous outbreaks and in a study from Italy during the COVID-19 pandemic^3,9^. In a recent study of Centers for Disease Control and Prevention, women were strongly advised to self-isolate regardless of quarantine periods^10^.

In this framework, pregnant women are reported to have higher levels of anxiety augmented by the protective measures taken relevant to perinatal care and labor. In most countries women are forced to separate from their supportive network due to a strict visitation policy system and present alone in the prenatal appointments and even in labor. Loss of social support and prolonged exposure of pregnant women to stressful events could be a leading pathway to postpartum depression (PPD)^11^.

PPD is defined as a major depression episode with perinatal onset; its prevalence ranges between 6.5% and 12.9%. Several studies regarding PPD in the pandemic have already been published^12-14^, and the first meta-analysis^15^ suggests that the pooled prevalence of PPD is 22%, representing a higher rate than before the pandemic^16^. Studies assessing PPD at 6 weeks postpartum revealed a prevalence of PPD up to 34%^17,18^.

According to a recent meta-analysis, the overall prevalence of perinatal anxiety has almost tripled during the COVID-19 pandemic from 15% to 40%^19,20^. In Greece, the reported levels of anxiety, as recorded during the first lockdown period, were >50% with a decreasing tendency towards the end of the restrictive measures reaching 34–40%, probably due to the efficacious control of the pandemic^21^.

Concerning PPD, however, to the best of our knowledge there are no studies assessing its prevalence during COVID-19 pandemic in Greece. Given this gap in the literature, we aim to assess the prevalence of PPD and anxiety symptoms in the Greek puerperal women and to evaluate the association of COVID-19 related concerns with antenatal anxiety and PPD symptoms.

## METHODS

### Study design and participants

This is a multicenter two-phase observational prospective cohort study conducted from June 2020 (one month after the first lockdown in Greece) to August 2020, involving 5 hospitals, throughout Greece. The study was approved by the ethics committees of all hospitals, and it was registered on clinicaltrials.gov. All procedures were in accordance with the World Medical Association Helsinki Declaration and signed informed consent was obtained from all participants.

In the first phase of the study, all parturients presenting for labor during June 2020, were asked to participate. Exclusion criteria were inability to read and write in Greek, history of an acute psychotic episode, intoxication or confusion. Each local investigator explained the aims of the study and a written informed consent was signed. The set of Phase 1 questionnaires (Generalized Anxiety Disorder-GAD-7 and a self-reported questionnaire about COVID-19 related concerns) was administered to all parturients. Demographic and medical variables were also recorded from the medical records. The second phase was conducted at 6–8 weeks postpartum by telephone and all women were asked to complete the Edinburgh Postnatal Depression Scale (EPDS). This is because PPD usually manifests at 6–8 weeks postpartum and in order to avoid the confounding impact of maternity blues (30–80% during the first 15 days postpartum). If a woman recorded suicidal ideation or a high score in the study tools, she was referred to a mental health service. Also, they were informed about the healthcare services and help lines operated during the pandemic.

### Measures and study instruments

Sociodemographic and pregnancy variables were collected through a questionnaire and medical records. COVID-19 related concerns were collected using a 38-items structured questionnaire grouped in 5 domains (pregnancy, family, adequacy of information, obstetrics/gynecology, and anesthetic concerns). Fourteen items were dichotomous (Y/N) or with multiple responses (e.g. ‘where did you get information regarding viral transmission to the baby’ with possible answers such as physician, media, social media, other) and 24 items were scored on a 10-point Likert scale (0–9, in general 0 refers to ‘not at all’ and 9 to ‘extremely’, e.g. how much are you concerned about your pregnancy due to pandemic?). The questionnaire was developed specifically for this study after careful examination of the available regarding common concerns and anxieties among pregnant women. Subsequently all items were reviewed by all authors based on their expertise, specialty, and empirical knowledge at the first wave of pandemic in Greece, and consensus was established^17-25^. Two parturients that were not included in the study sample were involved in this research by means of assisting in developing these research questions and in their prioritization in this structured questionnaire. Reliability analysis revealed a good strength of associations (Cronbach alpha=0.78).

PPD was assessed with the EPDS^26^, which is a 10-item self-report tool. Mothers were asked to check the response that is closest to how they have been feeling in the last 7 days in a 4-point Likert scale (0–3; score range: 0–30). PPD was defined as an EPDS score of ≥13. The reliability of the scale was high (Cronbach’s alpha=0.87).

Anxiety symptoms were assessed by the Greek version of GAD-7 which rates the frequency of anxiety symptoms in the last 2 weeks on a Likert scale ranging from 0–3 (‘not at all’ to ‘nearly every day’) with a standard cut-off point of ≥10 indicating moderate to severe anxiety symptoms^27^.

### Statistical analysis

All statistical analyses were performed using the Statistical Package for the Social Sciences (SPSS) version 26 for Mac. Summary statistics for all variables were calculated using means and standard deviation for continuous variables and frequency and proportions for categorical variables. The primary outcome variable was the EPDS score at 6–8 weeks postpartum. To assess the association of COVID-19 related concerns with anxiety and depressive symptoms, Pearson’s correlations tests were carried out, followed by partial correlations adjusted for age, demographic and obstetric variables. To assess the independent associations of COVID-19 related concerns with anxiety and depression, two separate binary logistic regression analyses were performed with dependent variable the GAD-7 score (<10 and ≥10) and EPDS score (<13 and ≥13), respectively. Independent variables in each analysis were the most significant associations of COVID-19-related concerns derived from the correlation analysis.

## RESULTS

### Demographic characteristics of the study sample

In total, 339 parturients enrolled in the study. There was a 2.65% dropout rate between the first and second phase of the study (9 participants were not located at 6 weeks). Finally, the sample consisted of 330 women with a mean age of 32.3 years (SD: 5.6) [maternal age <35, (n=207; 62.7%) and maternal age ≥35 (n=123; 37.3%)], at a gestational age of 38 weeks (SD: 1.9). The majority were married (n=300; 90.6%), 63.1% were employed (n=209) and 85.5% (n=282) had completed secondary education (high school). Labor was conducted via caesarean (n=211; 64%) or vaginal (n=119; 36%) delivery. Preterm deliveries accounted for 10% of the study sample (n=33) and term deliveries for 90% (n=297).

### Prevalence of postnatal depression and generalized anxiety disorder

In total, 43 mothers scored 13 or above in EPDS indicating that the prevalence of MDD (Major Depressive Disorder) was 13.2%. As far as anxiety symptoms are concerned, the mean score of GAD-7 before delivery was 6.3±5.0 indicating mild anxiety for parturients, while one-fourth (24.8%; n=82) could be identified as having probable, generalized anxiety disorder, when a cut-off point of ≥10 was used.

### Concerns about COVID-19

**Figure 1:**
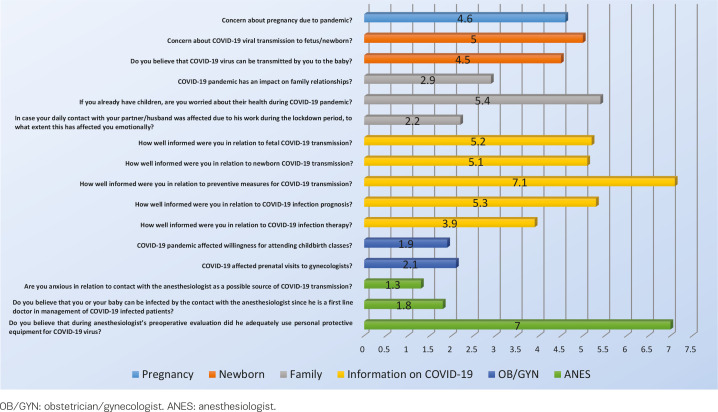
Questions (sorted in relation to pregnancy, newborn, family, information on COVID-19, obstetrics and anesthesiologist) related to concerns about COVID-19 and mean values

Figure 1 shows the mean scores for the items related to COVID-19 concerns. Concerns about pregnancy and pandemic and transmission to fetus/newborn were moderate. They also seemed quite worried about the health of older children (5.4±3.2). Moreover, antenatal care seemed mildly affected due to the pandemic; however, a proportion of 36% (n=119) reported a decrease in willingness to attend antenatal education courses and a similar proportion of 34% (n=112) reported fewer antenatal visits to an obstetrician/gynecologist than planned. Also, mild concerns about COVID-19 transmission were reported because of their contact with the anesthesiologist as a first line doctor.

### Correlations between COVID-19 concerns and anxiety symptoms

In univariate analysis (Table 1), a number of variables were associated with anxiety symptoms. A belief about transmission of the virus to the baby was positively correlated with anxiety score. Anxiety was significantly associated with the impact of COVID-19 outbreak on family relationships while the covariates did not affect this relationship. Parturients were anxious when their daily contact with their husband was affected by his work during the lockdown. As far as information related to transmission, our results revealed a negative relationship with anxiety, indicating that the better the information the less the anxiety. Finally, antenatal care as reflected in willingness to attend childbirth classes and prenatal visits was related to the GAD-7 total score. Partial correlation showed that certain associations were affected by the covariates like parturients’ age and gestational age (Table 1).

**Table 1:** Correlations among COVID-19 related concerns and anxiety symptoms (GAD-7)

*Questions*	*GAD-7 scale correlation to COVID-19 concern*					
	*No covariates*		*Controlled for mother’s age/gestational age*		*Controlled for mother’s age/gestational age marital status, type of delivery, high risk group, educational level*	
	*Pearson*	*p*	*Pearson*	*P*	*Pearson*	*P*
Concern about pregnancy due to pandemic?	0.098	0.075	0.154	0.031*	0.157	0.030*
Concern about COVID-19 viral transmission to fetus/newborn?	0.097	0.079	0.156	0.029*	0.151	0.037*
Do you believe that COVID-19 virus can be transmitted by you to the baby?	0.121	0.029*	0.114	0.113	0.116	0.111
COVID-19 pandemic has an impact on family relationships?	0.225	0.000***	0.210	0.003**	0.231	0.001**
If you already have children, are you worried about their health during COVID-19 pandemic?	0.121	0.077	0.155	0.030*	0.147	0.042*
In case your daily contact with your partner/husband was affected due to his work during the lockdown period, to what extent this has affected you emotionally?	0.156	0.006**	0.132	0.065	0.133	0.066
How well informed were you in relation to fetal COVID-19 transmission?	-0.164	0.003**	-0.136	0.058	-0.129	0.075
How well informed were you in relation to newborn COVID-19 transmission?	-0.150	0.006**	-0.146	0.042*	-0.140	0.053
How well informed were you in relation to preventive measures for COVID-19 transmission?	-0.103	0.061	-0.064	0.373	-0.046	0.526
How well informed were you in relation to COVID-19 infection prognosis?	-0.059	0.288	-0.071	0.327	-0.060	0.412
How well informed were you in relation to COVID-19 infection therapy?	-0.096	0.084	-0.149	0.037*	-0.141	0.052
COVID-19 pandemic affected willingness for attending childbirth classes?	0.146	0.008**	0.289	0.000***	0.309	0.000***
COVID-19 affected prenatal visits to gynecologists?	0.114	0.040*	0.140	0.051	0.152	0.035*
Are you anxious in relation to contact with the anesthesiologist as a possible source of COVID-19 transmission?	0.025	0.660	0.032	0.661	0.062	0.392
Do you believe that you or your baby can be infected by the contact with the anesthesiologist since he is a first line doctor in management of COVID-19 infected patients?	0.034	0.534	0.007	0.925	0.036	0.628
Do you believe that during anesthesiologist’s preoperative evaluation did he adequately use personal protective equipment for COVID-19 virus?	0.070	0.214	0.117	0.104	0.097	0.183

GAD-7: generalized anxiety disorder-7

* p<0.05

** p<0.01

*** p<0.001.

Separate univariate analysis (Table 2) showed that parturients aged ≥35 years were more anxious when they believed that the virus could be transmitted to the baby and less anxious when they were well informed about fetal transmission. The latter was also observed in parturients aged <35 years. Even so, younger women were more anxious if their daily contact with their husband was affected during the lockdown. In respect to gestational age, the anxiety symptoms were positively associated with their isolation from their partner due to his working during the lockdown only in preterm pregnancies. The same association was observed in term pregnancies along with the belief about viral transmission to the baby. Finally, in term pregnancies, a negative correlation was found between anxiety symptoms and the amount of information regarding the fetal transmission.

**Table 2:** Correlations among COVID-19 related concerns and anxiety symptoms (GAD-7) in advanced maternal age and preterm and term pregnancies

*Questions*	*Maternal age <35 (n=207)*			*Maternal age ≥ 35 (n=123)*			*Preterm (n=33)*			*Term (n=297)*		
	*Pearson*	*p*	*d*	*Pearson*	*p*	*d*	*Pearson*	*p*	*d*	*Pearson*	*p*	*d*
Do you believe that COVID-19 virus can be transmitted by you to the baby?	0.092	0.190	0.391	0.191	0.036*	0.389	0.190	0.297	0.387	0.116	0.047*	0.233
In case your daily contact with your partner/husband was affected due to his work during the lockdown period, to what extent this has affected you emotionally?	0.147	0.038*	0.297	0.178	0.061	0.362	0.382	0.028*	0.826	0.123	0.041*	0.248
How well informed were you in relation to fetal COVID-19 transmission?	-0.145	0.037*	-0.293	-0.197	0.030*	-0.402	-0.039	0.831	-0.078	-0.180	0.002**	-0.366
How well informed were you in relation to newborn COVID-19 transmission?	-0.149	0.032*	-0.301	-0.152	0.094	-0.307	-0.160	0.373	-0.324	-0.147	0.011*	-0.297
COVID-19 pandemic affected willingness for attending childbirth classes?	0.112	0.109	0.225	0.207	0.023*	0.423	0.456	0.008**	1.024	0.104	0.075	0.209
COVID-19 affected prenatal visits to gynecologists?	0.133	0.057	0.268	0.079	0.389	0.158	0.397	0.022*	0.865	0.086	0.139	0.172

GAD-7: generalized anxiety disorder-7

* p<0.05

** p<0.01

***p<0.001.

Subsequent regression analysis (Table 3) showed that the odds of being assessed with generalized anxiety disorder were higher in parturients who reported strong impact of pandemic on family relations (p=0.003, 95% CI: 1.060–1.316).

**Table 3:** COVID-19 related concerns independently associated with the maternal anxiety^*^

*Variables*	*OR (95% CI)*	*p*
**Demographics**		
Age	0.973 (0.901–1.050)	0.480
Advanced maternal age	1.146 (0.469–2.799)	0.765
Gestational age	1.065 (0.858–1.322)	0.566
Preterm/term	0.581 (0.154–2.201)	0.425
High risk group	1.442 (0.650–3.200)	0.368
Type of labor (NL/CS)	0.737 (0.403–1.349)	0.323
**COVID-19 related concerns**		
Belief about transmission to the baby	1.061 (0.959–1.174)	0.253
Family relations affected	1.181 (1.060–1.316)	0.003**
Emotional impact due to loss contact with husband	0.988 (0.892–1.095)	0.820
Information about transmission to the fetus	0.882 (0.716–1.088)	0.242
Information about transmission to the newborn	0.982 (0.807–1.196)	0.858
Affected willingness to childbirth classes	1.078 (0.983–1.183)	0.109
Affected prenatal visits to gynecologist	0.969 (0.879–1.069)	0.530

* Multivariable binary logistic regression analyses with dependent variable the ‘generalized anxiety disorder’ (GAD-7 ≥10) and independent variables the COVID-19 related concerns. The predictive values were calculated based on the probability of having ‘generalized anxiety disorder’ and the cut-off value between ‘case’ and ‘non-case’ was 0.500. Nagelkerke R^2^=0.129.

### Correlations between COVID-19 concerns and depressive symptoms at 6–8 weeks postpartum

Univariable analysis in Table 4 showed that different COVID-19 related variables correlated with EPDS depressive symptoms severity. When various variables were considered, only three remained significant. Depressive symptoms were positively associated with the degree of the emotional impact to the parturient due to the change in her daily contact with her husband because of his work in lockdown (p=0.018). Moreover, there were positive correlations with the degree of the anxiety related to her contact with the anesthesiologist as a possible source of COVID-19 transmission (p=0.043) as well as the belief that the anesthesiologist, as a first line doctor, could transmit the infection to her and the baby (p=0.023). Subsequent regression analysis (Table 5) showed that the odds of having a postnatal major depressive episode were higher only in parturients that reported higher levels of anxiety (p=0.000; 95% CI: 1.063–1.221).

**Table 4:** Correlations among COVID-19 related concerns and depressive symptoms at 6–8 weeks postpartum (EPDS)

*Questions*	*EPDS scale correlation to COVID-19 concern*							
	*No covariates*		*Controlled for mother’s age/gestational age*		*Controlled for mother’s age/gestational age marital status, type of delivery, high risk group, educational level*		*Controlled for mother’s age/gestational age marital status, type of delivery, high risk group, educational level and GAD-7*	
	*Pearson*	*p*	*Pearson*	*p*	*Pearson*	*p*	*Pearson*	*p*
Concern about pregnancy due to pandemic?	0.075	0.176	0.076	0.293	0.081	0.264	0.022	0.762
Concern about COVID-19 viral transmission to fetus/newborn?	0.074	0.186	0.043	0.555	0.046	0.528	-0.013	0.861
Do you believe that COVID-19 virus can be transmitted by you to the baby?	0.056	0.315	0.058	0.420	0.062	0.399	0.022	0.766
COVID-19 pandemic has an impact on family relationships?	0.209	0.000***	0.174	0.015*	0.177	0.014*	0.106	0.147
If you already have children, are you worried about their health during COVID-19 pandemic?	0.023	0.744	0.032	0.654	0.037	0.608	-0.020	0.787
In case your daily contact with your partner/husband was affected due to his work during the lockdown period, to what extent this has affected you emotionally?	0.219	0.000***	0.215	0.003**	0.206	0.004**	0.172	0.018*
How well informed were you in relation to fetal COVID-19 transmission?	-0.085	0.088	-0.068	0.346	-0.059	0.417	-0.016	0.829
How well informed were you in relation to newborn COVID-19 transmission?	-0.071	0.199	-0.046	0.521	-0.047	0.517	0.002	0.973
How well informed were you in relation to preventive measures for COVID-19 transmission?	-0.042	0.454	-0.015	0.837	-0.015	0.835	0.003	0.969
How well informed were you in relation to COVID-19 infection prognosis?	-0.069	0.212	-0.088	0.224	-0.086	0.236	-0.071	0.330
How well informed were you in relation to COVID-19 infection therapy?	-0.064	0.251	-0.053	0.466	-0.041	0.572	0.008	0.916
COVID-19 pandemic affected willingness for attending childbirth classes?	0.066	0.240	0.144	0.046*	0.149	0.040*	0.040	0.587
COVID-19 affected prenatal visits to gynecologists?	0.109	0.050*	0.053	0.464	0.054	0.462	-0.004	0.952
Are you anxious in relation to contact with the anesthesiologist as a possible source of COVID-19 transmission?	0.160	0.004**	0.147	0.041*	0.158	0.029*	0.147	0.043*
Do you believe that you or your baby can be infected by the contact with the anesthesiologist since he is a first line doctor in management of COVID-19 infected patients?	0.208	0.000***	0.166	0.021*	0.165	0.023*	0.165	0.023*
Do you believe that during anesthesiologist’s preoperative evaluation did he adequately use personal protective equipment for COVID-19 virus?	-0.033	0.563	-0.028	0.702	-0.042	0.563	-0.083	0.253

EPDS: Edinburg Postnatal Depression Scale, GAD-7: Generalized Anxiety Disorder-7

* p<0.05

** p<0.01

*** p<0.001.

**Table 5:** COVID-19 related concerns independently associated with PPD^*^

*Variables*	*OR (95% CI)*	*p*
**Demographics**		
Age	0.960 (0.867-1.062)	0.427
Advanced maternal age	0540 (0.167-1.751)	0.305
Gestational age	0.811 (0.624-1.053)	0.116
Preterm/term	4.334 (0.577-32.534)	0.154
High risk group	0.605 (0.197-1.859)	0.380
Type of labor (NL/CS)	1.182 (0.550-2.540)	0.669
**COVID-19 related concerns**		
Family relations affected	1.044 (0.917-1.189)	0.517
Emotional impact due to loss contact with husband	1.106 (0.984-1.242)	0.091
Anxiety to contact anesthesiologist	1.158 (0.931-1.441)	0.188
Baby infection by contact with anesthesiologist	0.977 (0.806-1.84)	0.814
GAD-7 score	1.139 (1.063-1.221)	0.000***

* Multivariable binary logistic regression analyses with dependent variable the ‘postpartum depression’ (EPDS ≥13) and independent variables the COVID-19 related concerns. The predictive values were calculated based on the probability of having ‘postpartum depression’ and the cut-off value between ‘case’ and ‘non-case’ was 0.500. Nagelkerke R^2^=0.178.

## DISCUSSION

### Main findings

In our study, the prevalence of PPD at 6 weeks postpartum was 13.2%. Data from Greece regarding the prevalence of PPD prior to the pandemic have revealed similar rates^28-30^. Gonidakis et al.^29^ found that the prevalence of PPD one month after delivery was 12.5% using the cut-off point of 12. Similarly, Koutra et al.^30^, using the same cut-off with our study (≥13), found that the prevalence of PPD was 13.6% at 8 weeks postpartum. Moreover, Leonardou et al.^28^ used the cut-off point of 11/12 and found that the prevalence of PPD was 12.4%. Vivilaki et al.^31^ in 2007–2008, in a sample of postpartum women, reported that 6.7% of mothers diagnosed with major depression, 11.7% with moderate and 31.7% with mild depression according to BDI-II scores. Therefore, the prevalence of PPD during the first phase of COVID-19 pandemic was not increased, most probably because Greece was not hit hard by the first pandemic wave. Confirmed cases during the study period (June 2020–August 2020) ranged from 2.937 on 1 June to 10.757 on 1 September; the latter represents a rate near to 1% of the total population. Moreover, the number of deaths were low compared to other countries (n=271 on 1 September 2020). Moreover, our study was conducted over a period where no strict restrictive measures were implemented. Although a number of studies report an increase in PPD, a recent meta-analysis showed that even though the EPDS score was higher in the pandemic compared to the nonpandemic period, it did not reach the level of statistical significance^12-14,32^. In contrast, parturients reported high antenatal levels of anxiety, since 24.8% of them scored ≥10 in GAD-7. A recent meta-analysis, before the pandemic showed that self-reported anxiety symptoms prevalence was 24.6% in the third trimester and the overall prevalence of any anxiety disorder was 15.2%^19^. During the pandemic, two studies using GAD-7 reported lower rates compared to our study, of moderate anxiety (14% and 21.6%) in countries hit hard by COVID-19^13,33^. Similarly, in a multinational study of 3097 pregnant women, 11% scored ≥10 in GAD-7^13^. Exception to these rates is the study of Preis et al.^33^ which revealed substantial levels of moderate to severe anxiety up to 43.3%. Evidence suggests that prenatal anxiety increases with the severity of the measures imposed. Interestingly, our recruitment was undertaken in a period where strict restrictive measures were not applied since Greece was not hit so hard in the first pandemic wave. One explanation is that family and social support during pregnancy in Greek families is very high and this was disrupted during lockdown, right before recruitment. Additionally, parturients were not allowed to have companions in the antenatal visits and in the hospital before delivery. By tradition, in Greece parturients have frequent visits to the hospital, and social support is associated with increased mental health and emotional well-being^34^. Still, we cannot ignore that anxiety measurement (even if GAD refers to the last 2 weeks) was undertaken right before delivery and this per se represents a stressful moment.

Our findings suggest that parturients were more anxious when they believed that SARS-CoV-2 virus could be transmitted by them to their baby. Anxiety could be due to lack of solid scientific evidence about transplacental transmission at the time the study was conducted^35^. However, even if the evidence was against transmission, parturients could be influenced by the so-called infodemic phenomenon. Moreover, antenatal care was correlated with GAD-7 scores probably because of the fear of transmission. Finally, anxiety symptoms were associated with the decreased contact with the partner, probably due to lack of emotional support.

In our study, anxiety of advanced maternal age mothers was correlated with the belief that the virus could be transmitted to the baby, something that was not observed in the younger group. Advanced maternal age and the fear of related complications in pregnancy could be the explanation. In contrast, younger mothers were anxious when they have lost their daily contact with their husband. Anxiety of preterm pregnancy mothers (in contrast with term pregnancies) is not associated with beliefs about transmission to the baby probably because their primary source of anxiety is the survival or the complications to the newborn due to the preterm pregnancy itself^36,37^. In general, little is known about predictive risk factors for anxiety disorders in pregnancy and postnatal period, being a neglected area of research^38-42^. In our study during the pandemic, parturients being at higher risk for generalized anxiety disorder were those whose family relations were affected the most. This is indicative of the importance of marital and social support during pregnancy that a woman calls on during stressful events^43^.

Depressive symptoms were correlated with various COVID-19 concerns. The PPD was positively associated with the reduced daily contact with the husband and with the fear that anesthesiologists could be a possible source of transmission. Previous studies related to SARS outbreak showed that the relationship with the partner was compromised due to interruption of intimacy and fear of transmission^34,44^. Additionally, women who experience higher levels of support from their partners during the second trimester of pregnancy present lower levels of psychological implications postnatally^44^. Therefore, it comes as no surprise that COVID-19 outbreak preventive measures related to partners’ distancing implemented in our study population could predispose to PPD. Moreover, anesthesiologists who come in close contact with severely ill patients are exposed to airborne viral transmission and are among the medical professionals of highest risk of infection^45^. Furthermore, their contribution to the current pandemic management is vastly praised by the social media and the public^46^. Considering that the public is overexposed to social media and TV news as a source of information during this pandemic^47^, parturients were directly exposed to the infodemic phenomenon. However, concerning predictive factors, in our study only high levels of anxiety were associated with postnatal depression, a finding consistent with the literature^48-50^.

### Limitations

Our study has some limitations that need to be addressed. One lies in its cross-sectional nature. Yet it involves 5 centers throughout Greece (three located in Athens where half of the total population lives). Moreover, it assesses depressive symptoms at 6 weeks postpartum in order to avoid the ‘baby blues’ period. Although we used the most common PPD tool, we did not confirm the diagnosis using a structured interview. Another limitation is the fact that the 6 weeks’ postpartum assessment was performed by telephone contact where women underreport their symptoms. However, there are reports that EPDS administration over the telephone has high reliability^51^. Finally, the study was conducted during the first pandemic wave where COVID-19 confirmed cases and deaths were not high in Greece. However, to the best of our knowledge, this is the first study in Greece assessing the prevalence of PPD during the COVID-19 pandemic.

### Future research

Unfortunately, the pandemic is still on the rise and new mutations appear that may be a trigger for psychological distress for the population and of course for pregnant women. Future longitudinal research is needed to better identify high risk parturients and to provide the best maternal and child care both prenatal and postnatal. Future research should focus on psychosocial features such as the general support to pregnant women, e.g. marital and social support during pregnancy as well as medical staff support just after the labor, with well validated tools. Finally, since countries do not have the same infection rate throughout their region (and in different times this was the case in Greece), it is important to conduct further prevalence and longitudinal studies (throughout pregnancy) with larger samples in different regions to identify the effect of covid-infection rate in maternal psychological distress.

## CONCLUSIONS

PPD rates according to our results did not increase during the first phase of the pandemic in Greece. It is known that many people do not develop psychopathology soon after a traumatic event and this may be the case in our sample. On the other hand, anxiety levels seem to be increased, indicating that there is a need for close monitoring and anxiety screening in order to identify women who need support in the pandemic era. Maternity care providers should employ an informative well-structured antenatal program in respect to the parturients’ concerns related to this pandemic. If restrictive measures are implemented, telephone contacts, video calls with the specialists or online antenatal courses could be applied. In such antenatal programs, enhancement of social support of pregnant women (e.g. involvement of husband and other close relatives) should be included. It is important to call attention to the need for close monitoring of antenatal care during the pandemic since a significant proportion of women were forced to skip the usual prepartum care appointments, like in previous outbreaks. Decreased antenatal care not only poses a medical danger to the mother and the fetus but also relates to PPD symptoms. Additionally, anesthesiologists should address parturients concerns. Safe emotional contact with their partners should be always advocated. Finally, a follow-up using teleconference in the first weeks of labor should be established by midwives in order to identify symptoms of PPD^52^, especially in those with high antenatal anxiety which should be screened during pandemic in pregnancy as well as before labor.

## Data Availability

The data supporting this research are available from the authors on reasonable request.

## PROTOCOL REGISTRATION

Clinicaltrials.gov: NCT04437342. Submitted on 15.06.2020 Official Title: Postpartum Depression in the Covid-19 Pandemic and the Impact of Anaesthesia
